# Liver Viscosity Imaging and Disease Severity in Alcohol-Associated Hepatitis: A Prospective Cross-Sectional Pilot Study

**DOI:** 10.3390/diagnostics16193128

**Published:** 2026-09-26

**Authors:** Carmen-Georgeta Fierbințeanu-Braticevici, Priscila Mădălina Ologeanu, Ionuț-Cristian Costin, Andrei Cozea, Vlad-Teodor Enciu

**Affiliations:** 1 Gastroenterology and Internal Medicine II Department, “Carol Davila” University of Medicine and Pharmacy, 050474 Bucharest, Romania; 2 ENT Department, Carol Davila University of Medicine and Pharmacy, 020021 Bucharest, Romania

**Keywords:** liver viscosity, Sound Touch Visco-elastography Imaging, alcohol-associated hepatitis, multiparametric ultrasound, alcoholic hepatitis disease severity, liver stiffness

## Abstract

**Background:** Alcohol-associated hepatitis (AH) is an acute inflammatory phenotype of alcohol-associated liver disease associated with substantial short-term mortality. Established clinical scores quantify disease severity but do not directly characterize liver tissue mechanics. Ultrasound-derived viscosity-related measurements may provide complementary, non-invasive information on viscoelastic tissue behavior, although their biological determinants remain incompletely defined. **Objective:** We aimed to evaluate the association between liver viscosity and AH through the Sound Touch Visco-elastography Imaging (STVi) parameter and inflammatory, biochemical, and MDF-defined severity measures, and to explore its discriminatory performance. **Methods:** This prospective, single-center, cross-sectional pilot study included 62 patients with clinically diagnosed AH and 30 healthy volunteers. Participants underwent multiparametric ultrasound assessment using the Resona A20 platform with STVi, liver stiffness measurement, and attenuation imaging. **Results:** The viscosity-related parameter was higher in patients with AH than in healthy volunteers and was associated with liver stiffness, inflammatory markers, and MDF-defined severity. The exploratory AH-versus-healthy comparison yielded an AUC of 0.873 (95% CI, 0.797–0.934). Within the AH cohort, 28 patients had severe AH (MDF ≥ 32) and 34 had non-severe AH. For MDF-defined severe AH, the AUC was 0.863 (bootstrap 95% CI, 0.756–0.955). At the Youden-derived cutoff of 2.8 Pa·s, sensitivity was 78.6% and specificity was 94.1%. **Conclusions:** STVi provides a model-derived, platform-dependent viscosity-related parameter associated with inflammatory measures and clinical severity in AH. These preliminary findings suggest that liver viscosity imaging may represent a valuable new avenue for the non-invasive assessment of AH. However, standardized acquisition, longitudinal assessment, histological correlation, and external validation are required before clinical implementation.

## 1. Introduction

Excessive alcohol consumption is a major cause of preventable morbidity and mortality [1]. Alcohol-associated liver disease (ALD) encompasses a spectrum ranging from steatosis to alcohol-associated hepatitis (AH) and cirrhosis [2,3]. AH is an acute inflammatory syndrome characterized by recent-onset jaundice and liver dysfunction in the setting of sustained heavy alcohol consumption. Diagnosis is usually clinical, using standardized criteria and exclusion of competing causes, while liver biopsy is reserved for diagnostically uncertain cases [4,5]. Severe AH is associated with substantial short-term mortality, making early and accurate severity assessment clinically important [6,7].

Current severity scores are based primarily on laboratory indicators of hepatic dysfunction and systemic illness. Although these tools remain central to clinical assessment, they do not directly characterize the mechanical tissue changes in AH. Conventional liver stiffness measurements are influenced not only by fibrosis but also by inflammation, cholestasis, congestion, and other acute changes, which may complicate their interpretation in AH. Contemporary multiparametric ultrasound approaches emphasize a multiparametric ultrasound framework in which elastography and quantitative attenuation measurements are interpreted with attention to biological and technical confounders [8,9]. A quantitative parameter obtained during the same bedside ultrasound examination could therefore provide complementary tissue-mechanical information [10,11].

Sound Touch Visco-elastography Imaging (STVi) evaluates the frequency-dependent propagation of shear waves in viscoelastic tissue and derives a viscosity-related coefficient through model fitting [12]. The resulting value, expressed in Pa·s, is a model-derived viscosity-related coefficient reflecting the dissipative component of tissue mechanical behavior. Viscosity-related ultrasound measurements have been investigated in healthy individuals and heterogeneous chronic liver disease populations, but evidence specific to AH remains limited. Consequently, their relationships with inflammation, background structural liver disease, and established AH severity measures require focused evaluation.

The primary aim of this study was to evaluate the cross-sectional association between the STVi viscosity-related parameter and MDF-defined severity in patients with AH. Secondary aims were to examine its relationships with inflammatory, biochemical, attenuation, and liver stiffness parameters and to explore its ability to discriminate severe from non-severe AH. The study was designed as an exploratory pilot investigation and was not intended to establish histological validation, prognostic utility, or superiority over conventional liver stiffness measurement.

## 2. Materials and Methods

### 2.1. Study Design, Setting, and Participants

This prospective, single-center, cross-sectional pilot study was conducted at the Gastroenterology Department of the Emergency University Hospital Bucharest. Adult patients hospitalized with clinically diagnosed alcohol-associated hepatitis were assessed for eligibility between February 2024 and December 2025. Patients fulfilling established clinical criteria for probable AH and without a competing explanation for the acute liver injury and jaundice were included. Clinical evaluation, blood sampling, disease-severity assessment, and the index ultrasound examination were performed during the same hospitalization, at admission.

### 2.2. Diagnostic Criteria for Alcohol-Associated Hepatitis

- Jaundice with onset within the preceding 8 weeks;
- Sustained alcohol consumption of >40 g/day for women or >60 g/day for men for at least 6 months, with <60 days of abstinence before the onset of jaundice;
- Aspartate aminotransferase (AST) > 50 U/L, an AST/alanine aminotransferase (ALT) ratio > 1.5, and both AST and ALT < 400 U/L;
- Total bilirubin ≥ 3 mg/dL.

### 2.3. Exclusion Criteria

- Possible ischemic hepatitis, suggested by severe upper gastrointestinal bleeding, hypotension, or cocaine use within 7 days before symptom onset;
- Possible inherited or metabolic liver disease, including Wilson disease or alpha-1 antitrypsin deficiency;
- Possible drug-induced liver injury, defined by exposure to a potentially causative medication within 30 days before jaundice onset;
- Uncertain alcohol-use history or atypical laboratory findings, including AST < 50 U/L or >400 U/L, an AST/ALT ratio < 1.5, antinuclear antibody titer > 1:160, or anti-smooth muscle antibody titer > 1:80.

Alternative causes of acute liver injury and jaundice were systematically evaluated during the index hospitalization. The diagnostic work-up included clinical history and examination, medication exposure, routine laboratory testing, viral hepatitis serology, autoimmune serology when clinically indicated, and conventional abdominal ultrasound. Viral hepatitis was excluded by negative serological testing, and biliary obstruction was excluded by the absence of biliary dilatation or other obstructive findings on ultrasound. Possible ischemic hepatitis was assessed in relation to hypotension, severe gastrointestinal bleeding, or recent cocaine use, whereas drug-induced liver injury was evaluated according to recent exposure to potentially hepatotoxic medications. Active infection/sepsis and clinically significant cardiac congestion were assessed as part of the routine inpatient clinical and laboratory evaluation.

### 2.4. Healthy Control Group

During the same study period, healthy volunteers undergoing routine medical evaluation were recruited as an exploratory control group. Controls reported no current alcohol consumption, had no relevant medical history or ongoing pharmacological treatment, and had liver-related laboratory parameters within the reference ranges. Viral hepatitis screening was negative, conventional liver ultrasound findings were normal, and participants had no obesity or clinically evident metabolic disease. Controls were recruited from eligible volunteers and were not individually matched to patients with alcohol-associated hepatitis. The comparison with healthy volunteers was undertaken as an exploratory proof-of-concept analysis to determine whether liver viscosity differed between patients with AH and individuals without clinically evident liver disease.

### 2.5. Clinical, Laboratory, and Severity Assessment

Clinical evaluation and blood sampling were performed during the index hospitalization. Laboratory assessment included complete blood count, C-reactive protein (CRP), erythrocyte sedimentation rate (ESR), ALT, AST, total bilirubin, alkaline phosphatase, gamma-glutamyl transferase, prothrombin time, and international normalized ratio. Biological and ultrasound assessments were performed on the same day.

The Maddrey discriminant function (MDF) was calculated for patients with AH using the following formula: MDF = 4.6 × (patient prothrombin time − control prothrombin time) + total bilirubin (mg/dL). An MDF ≥ 32 was used to define severe AH.

### 2.6. Multiparametric Ultrasound Assessment

Liver ultrasound examinations were performed using the Resona A20 ultrasound system (Shenzhen Mindray Bio-Medical Electronics Co., Ltd., Shenzhen, China), equipped with the Sound Touch Visco-elastography Imaging (STVi) application and the SC7-1U multifrequency convex-array transducer (1–7 MHz). Parallel multiparametric assessment quantified shear-wave-derived liver stiffness (kPa), a model-derived viscosity-related coefficient (Pa·s), and the ultrasound attenuation coefficient (dB/cm/MHz).

Examinations were performed through a right intercostal approach, with the participant in the supine position and during a brief breath-hold. The acquisition box was positioned within homogeneous liver parenchyma, avoiding large vessels, bile ducts, focal lesions, and imaging artifacts. A system-generated circular region of interest with a diameter of 13 mm was placed approximately 4–5 cm below the skin surface within the most homogeneous area of the color-coded map. An acquisition was considered technically acceptable when the motion-stability index displayed five green stars and the reliability map was homogeneous, with an RLB index of at least 90%. Ten technically valid acquisitions were obtained for each participant, and the median value was used for analysis. The system automatically generated the interquartile range, IQR-to-median ratio, mean, standard deviation, and SD-to-mean ratio (Figure 1 and Figure 2). Technical failure was defined as the inability to obtain ten valid acquisitions fulfilling the predefined stability and reliability criteria after repeated attempts. No technical failures occurred.

All ultrasound examinations were performed by a senior hepatologist with 40 years of experience in abdominal ultrasonography. At the time of image acquisition and interpretation, the operator was blinded to the participants’ laboratory results, MDF and formal study-group allocation.

### 2.7. Ethical Considerations

The study was conducted in accordance with the Declaration of Helsinki and was approved by the Ethics Committee of the Emergency University Hospital Bucharest (approval no. 3110, 16 January 2024). Written informed consent was obtained from all participants in both groups before study procedures.

### 2.8. Statistical Analysis

Data were entered into Microsoft Excel and analyzed using Python version 3.13.5 with the pandas, scipy, matplotlib, and statsmodels libraries. Distributional characteristics of continuous variables were assessed by graphical inspection and the Shapiro–Wilk test. Because several imaging and laboratory variables showed non-normal distributions, continuous data are presented as median [interquartile range] and between-group comparisons were performed using the Mann–Whitney U test. Categorical variables were compared using the chi-square test when applicable.

Associations between continuous variables were evaluated using Spearman rank correlation coefficients. To limit overfitting, the multivariable analysis of the viscosity-related parameter was restricted to a parsimonious clinically prespecified linear regression model including liver stiffness and C-reactive protein (CRP), representing tissue-mechanical and inflammatory domains, respectively. Multicollinearity was assessed using variance inflation factors (VIFs), and model fit was summarized using R^2^ and adjusted R^2^. Residual normality, heteroscedasticity, and influential observations were evaluated using the Shapiro–Wilk test, the Breusch–Pagan test, and Cook’s distance. Because heteroscedasticity was detected, HC3 heteroscedasticity-consistent standard errors were used for inference.

Receiver operating characteristic (ROC) curves were generated for the exploratory comparison of AH versus healthy controls and for discrimination of MDF-defined severe versus non-severe AH. The severe-AH analysis was restricted exclusively to the 62-patient AH cohort. Candidate thresholds were selected by maximizing the Youden index. AUC uncertainty was assessed using 5000 stratified bootstrap resamples, while exact binomial confidence intervals were used for sensitivity, specificity, positive predictive value (PPV), and negative predictive value (NPV). PPV and NPV were interpreted as prevalence-dependent cohort estimates. Given the exploratory pilot design and the number of statistical comparisons, no formal multiplicity adjustment was applied and individual *p*-values should be interpreted cautiously. No formal a priori sample-size calculation was performed; enrollment was feasibility-based over the prespecified recruitment period. All tests were two-sided, with *p* < 0.05 considered statistically significant.

## 3. Results

### 3.1. Cohort Characteristics

The study included 92 participants: 62 patients with AH and 30 healthy volunteers. The final analytic cohort comprised all participants included during the study period with technically valid ultrasound measurements and the variables required for the reported analyses. No technical failures occurred. Descriptive imaging and laboratory characteristics are presented in Table 1.

### 3.2. Characteristics According to MDF-Defined Severity

Within the AH cohort, 28 patients were classified as severe (MDF ≥ 32) and 34 as non-severe. Severe AH was characterized by higher median viscosity-related values, liver stiffness, CRP, ESR, AST, bilirubin, INR, prothrombin time, and alkaline phosphatase among the available analytical variables, whereas attenuation coefficient, white blood cell count, ALT, and GGT did not differ significantly between severity groups. These subgroup comparisons are exploratory and are presented in Table 2.

### 3.3. Correlation Analysis

Spearman analysis showed positive associations of the viscosity-related parameter with liver stiffness (ρ = 0.67), MDF (ρ = 0.66), CRP (ρ = 0.63), INR (ρ = 0.63), and prothrombin time (ρ = 0.63). More modest associations were observed with ESR and AST. Correlations with attenuation coefficient, white blood cell count, ALT, and total bilirubin were weak and not statistically significant. The complete results are presented in Table 3.

### 3.4. Multivariable Analysis of the STVi Viscosity-Related Parameter

A clinically prespecified model including liver stiffness and CRP explained 65.0% of the variance in the viscosity-related parameter (adjusted R^2^ = 0.638). Multicollinearity was negligible (VIF = 1.01 for both predictors). Because the Breusch–Pagan test indicated heteroscedasticity (*p* < 0.001), HC3 robust standard errors were used. Both liver stiffness and CRP remained associated with the viscosity-related parameter using robust inference. Residuals did not show a significant departure from normality (Shapiro–Wilk *p* = 0.091). One observation (OIV) had the largest Cook’s distance (0.45); exclusion of this observation in sensitivity analysis did not alter the direction or statistical significance of either association (Table 4).

### 3.5. Exploratory Receiver Operating Characteristic Analysis

The comparison between patients with AH and the selected healthy volunteer group was interpreted as an exploratory proof-of-concept analysis rather than a clinically representative diagnostic-accuracy assessment. Liver viscosity yielded an AUC of 0.873 (95% CI, 0.797–0.934). At the candidate cutoff of 1.8 Pa·s, sensitivity was 66.1% and specificity was 96.6% (Figure 3 and Table 5).

Within the 62-patient AH cohort, 28 patients had severe AH and 34 had non-severe AH. The AH ROC analysis yielded an AUC of 0.863 (bootstrap 95% CI, 0.756–0.955). The threshold maximizing the Youden index was 2.8 Pa·s. At this threshold, sensitivity was 78.6%, specificity 94.1%, PPV 91.7%, and NPV 84.2%. The corresponding raw classification counts were 22 true positives, 6 false negatives, 32 true negatives, and 2 false positives (Figure 4 and Table 6).

### 3.6. Exploratory Comparison with Liver Stiffness and CRP

For MDF-defined severe AH, the AUC of the viscosity-related parameter (0.863) was numerically higher than the AUCs of liver stiffness (0.758) and CRP (0.809) (Table 7).

## 4. Discussion

In this prospective cross-sectional pilot study, the STVi-derived viscosity-related parameter differed between patients with AH and healthy volunteers and was associated with inflammatory markers, liver-related laboratory parameters, liver stiffness, and established measures of AH severity. Within the AH cohort, liver viscosity also showed promising discriminatory performance for MDF-defined severe AH (AUC 0.863). In line with current EASL and AASLD guidelines, which highlight the limitations of liver biopsy and encourage the development and validation of non-invasive assessment tools, our findings support further investigation of liver viscosity as a potentially valuable complementary biomarker for inflammatory activity and disease severity in AH [13,14,15].

AH is an acute inflammatory syndrome that frequently develops on a background of chronic ALD and may be associated with substantial short-term mortality. Current guidelines support a primarily clinical diagnosis based on compatible alcohol exposure, recent-onset jaundice, characteristic laboratory abnormalities, and exclusion of alternative causes, with liver biopsy generally reserved for cases of diagnostic uncertainty [4,5,14]. Although liver stiffness measurement is widely used in chronic liver disease, its interpretation in AH is complicated by inflammation, cholestasis, and congestion, which may increase liver stiffness independently of fibrosis [8,16,17]. Moreover, because AH may occur on a background of pre-existing fibrosis or cirrhosis, separating acute inflammatory effects from chronic structural changes remains challenging. These limitations provide a rationale for investigating additional tissue-mechanical parameters.

Using the Resona A20 platform, we performed a multiparametric examination incorporating attenuation imaging, liver stiffness measurement, and STVi. This approach provides several quantitative parameters during a single examination. In viscoelastic media, shear-wave propagation is frequency dependent and energy is dissipated during propagation. STVi uses this dispersion behavior and viscoelastic model fitting to derive a viscosity-related coefficient. The present study focused on whether this parameter was associated with clinical severity and inflammatory activity. The measurements were performed according to the protocol of a pilot study using multimodal imaging, including B-mode morphology, elastography, and viscoelastic assessment of liver tissue [12]. The STVi-derived viscosity parameter may be influenced by fibrosis, inflammatory changes, steatosis, and tissue heterogeneity, as well as by technical and model-related factors [12,16,17]. The exploratory comparison with healthy volunteers suggested that liver viscosity may distinguish patients with AH from an apparently healthy population. The observed direction of the group difference is consistent with previous reports showing higher viscosity-related measurements in chronic liver disease than in healthy controls [18]. However, because the control group was highly selected and not matched to the AH cohort, this comparison should be interpreted as an exploratory proof-of-concept analysis rather than as evidence of clinically applicable diagnostic accuracy.

A readily available marker that adds information to established severity scores would be clinically relevant. In the present study, the STVi viscosity-related parameter was positively associated with MDF-defined disease severity and with inflammatory markers, including C-reactive protein. For discrimination of MDF-defined severe AH, STVi yielded an AUC of 0.863, compared with 0.758 for liver stiffness and 0.809 for CRP. Although the AUC for STVi was numerically higher, this pilot study does not establish statistical superiority or clinically meaningful incremental value over conventional liver stiffness or established clinical scores. However, the present cross-sectional data cannot determine whether viscosity improves clinical decision-making, predicts short-term mortality, identifies candidates for corticosteroid therapy, or predicts treatment response. These potential applications require prospective outcome-based studies.

The relationship between STVi and conventional liver stiffness is particularly important. Liver stiffness predominantly reflects the elastic component of the shear-wave response, whereas viscosity-related or dispersion parameters attempt to characterize the frequency-dependent dissipative component [19,20,21]. These components are not biologically independent, and both may be influenced by overlapping pathological processes. In the present study, liver stiffness remained independently associated with the STVi viscosity-related parameter in the multivariable model. STVi should therefore be considered a potentially complementary tissue-mechanical parameter rather than a replacement for conventional elastography.

Evidence regarding liver viscosity in AH remains limited, and most published studies have included heterogeneous chronic liver disease populations [18,22,23]. Some studies have reported associations between viscosity-related measurements and necro-inflammatory activity [22], whereas others found stronger relationships with fibrosis than with inflammation [11,23]. These differences may partly reflect variations in the underlying pathological substrate. AH is typically characterized by hepatocyte ballooning, lobular inflammation, and neutrophilic infiltration [24,25]. These changes, together with tissue edema, increased cellularity, and altered tissue fluid content, may plausibly increase energy dissipation during shear-wave propagation and contribute to higher viscosity-related measurements. In contrast, chronic structural liver disease is characterized by fibrosis, collagen deposition, and tissue remodeling, which predominantly affect the elastic component of tissue behavior [26]. Even when AH develops on a background of chronic alcohol-associated liver disease, the acute inflammatory response remains predominantly neutrophil-driven. Nevertheless, because liver biopsy was not performed in the present study, we cannot determine the relative contribution of acute inflammation and chronic structural changes to the observed STVi signal, nor can STVi-derived viscosity be considered a validated surrogate of histological necro-inflammatory activity.

The attenuation coefficient was higher in patients with AH than in healthy controls. Quantitative attenuation is an established ultrasound-derived parameter for the assessment of hepatic fat content, and current WFUMB guidance emphasizes standardized acquisition and consideration of biological and technical factors [9]. In the present study, attenuation was not significantly associated with the STVi viscosity-related parameter, suggesting that the observed viscosity signal was not simply explained by coexisting hepatic steatosis. Similar findings have been reported in a cohort of 120 patients with heterogeneous chronic liver diseases, in which liver viscosity was associated with fibrosis rather than steatosis [27]. Although steatosis and related microstructural changes may influence tissue mechanical behavior, hepatic fat content does not necessarily parallel inflammatory burden or disease severity. In advanced liver disease, a reduction in hepatic fat content with disease progression, sometimes referred to as “burning-out” steatosis, has also been described [28].

More broadly, viscosity-related and shear-wave dispersion techniques are not standardized across manufacturers, and prior studies have used different viscoelastic parameters and processing approaches [19,20,21]. For instance, Canon Shear Wave Dispersion directly quantifies the slope of the relationship between shear-wave speed and frequency. Because the measured quantity is the change in propagation velocity per unit change in frequency, it is expressed in (m/s)/kHz [21,29]. This dispersion slope is therefore a viscosity-related parameter rather than a direct viscosity coefficient. In contrast, STVi analyzes the frequency-dependent shear-wave behavior through viscoelastic model fitting and derives a viscosity-related coefficient expressed in Pa·s, the SI unit of dynamic viscosity [12]. Thus, the difference in units reflects a fundamental difference in the mathematical output: Canon reports the slope of the dispersion curve, whereas STVi reports a model-derived estimate of its viscous component. Consequently, values and thresholds obtained with the two approaches cannot be directly converted or considered interchangeable because they depend on the underlying model, frequency range, acquisition method, and manufacturer-specific processing algorithms. This reinforces the need for platform-specific validation and standardized acquisition protocols [12,19,21,29].

Several limitations should be acknowledged. This was a single-center, cross-sectional pilot study with a limited sample size and no external validation. The cross-sectional design and absence of longitudinal follow-up prevented assessment of short-term mortality, treatment response, or prognostic utility. Liver biopsy was not available as a reference standard for necro-inflammatory activity; therefore, the relationship between the STVi viscosity-related parameter and histological inflammation cannot be directly established. In addition, detailed characterization of background fibrosis and chronic structural liver disease was not systematically available, limiting separation of their contribution from that of acute inflammatory changes. Intraobserver and interobserver reproducibility were not assessed. Finally, viscosity-related ultrasound measurements are not yet standardized across platforms, and the internally derived cutoffs and diagnostic-performance estimates require confirmation in larger, clinically representative, multicenter cohorts.

If validated in larger prospective cohorts, STVi could provide complementary information as part of a routine multiparametric ultrasound examination. Future studies should evaluate longitudinal changes in viscosity-related measurements during clinical recovery and their relationship with disease activity. AI-enabled multiparametric models integrating STVi with liver stiffness, attenuation, conventional imaging features, laboratory data, and clinical variables may also represent a promising direction for future research [30].

## 5. Conclusions

In this prospective cross-sectional pilot study, the STVi-derived viscosity-related parameter was associated with inflammatory markers and MDF-defined disease severity in alcohol-associated hepatitis. Liver viscosity imaging may therefore represent a valuable new component of multiparametric ultrasound assessment in AH, with the potential to provide complementary information beyond conventional liver stiffness. Further prospective and multicenter studies are needed to clarify its clinical utility and role in disease stratification.

## Figures and Tables

**Figure 1:**
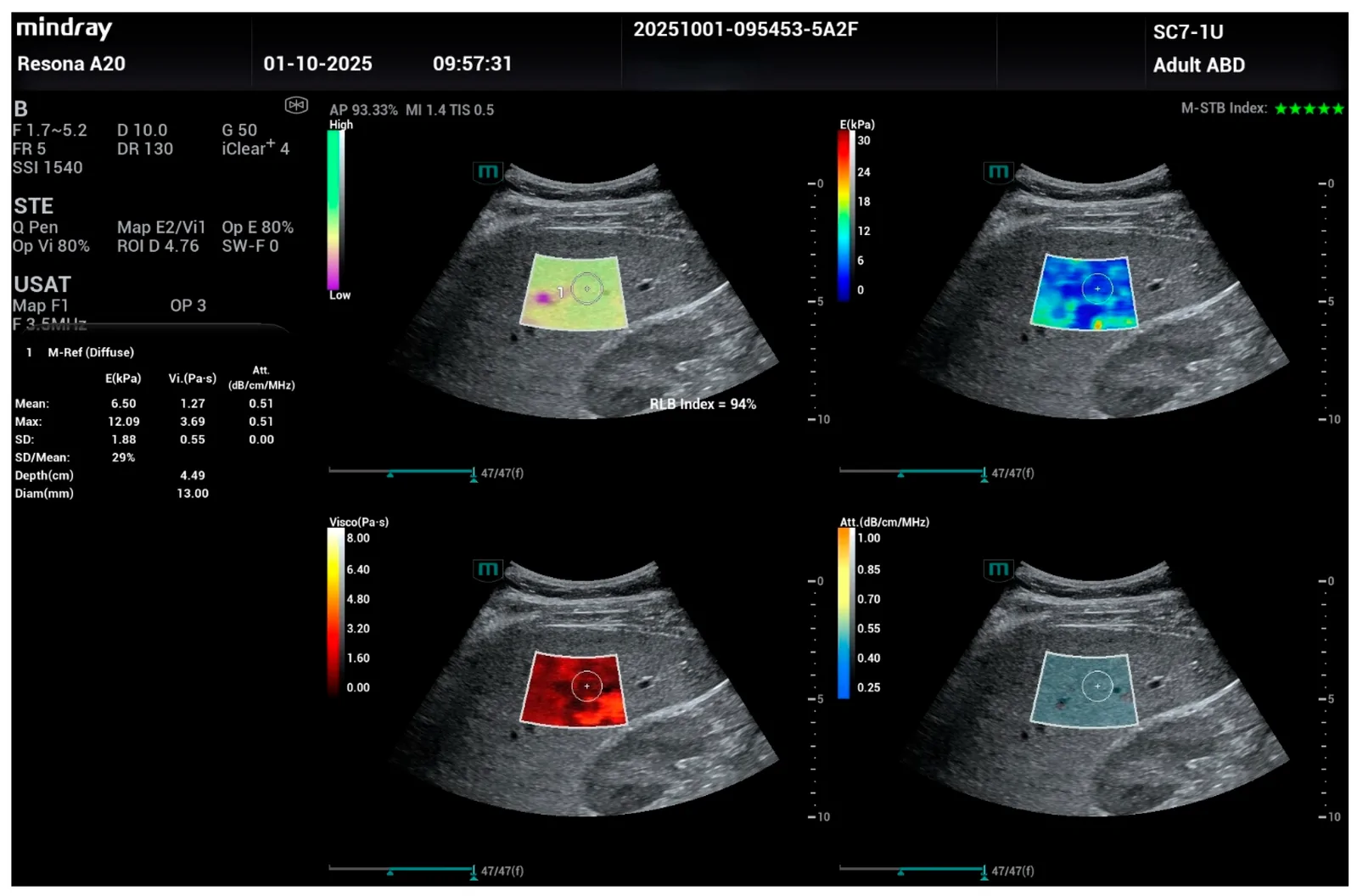
Representative multiparametric ultrasound assessment of liver tissue using the Resona A20 system. The examination simultaneously displays B-mode imaging and quantitative color-coded maps for liver stiffness, expressed in kPa; liver viscosity, expressed in Pa·s; and ultrasound attenuation, expressed in dB/cm/MHz. The circular measurement region of interest was positioned within homogeneous liver parenchyma while avoiding major vessels and bile ducts. The motion-stability indicator (M-STB Index) and reliability map were used to assess acquisition quality.

**Figure 2:**
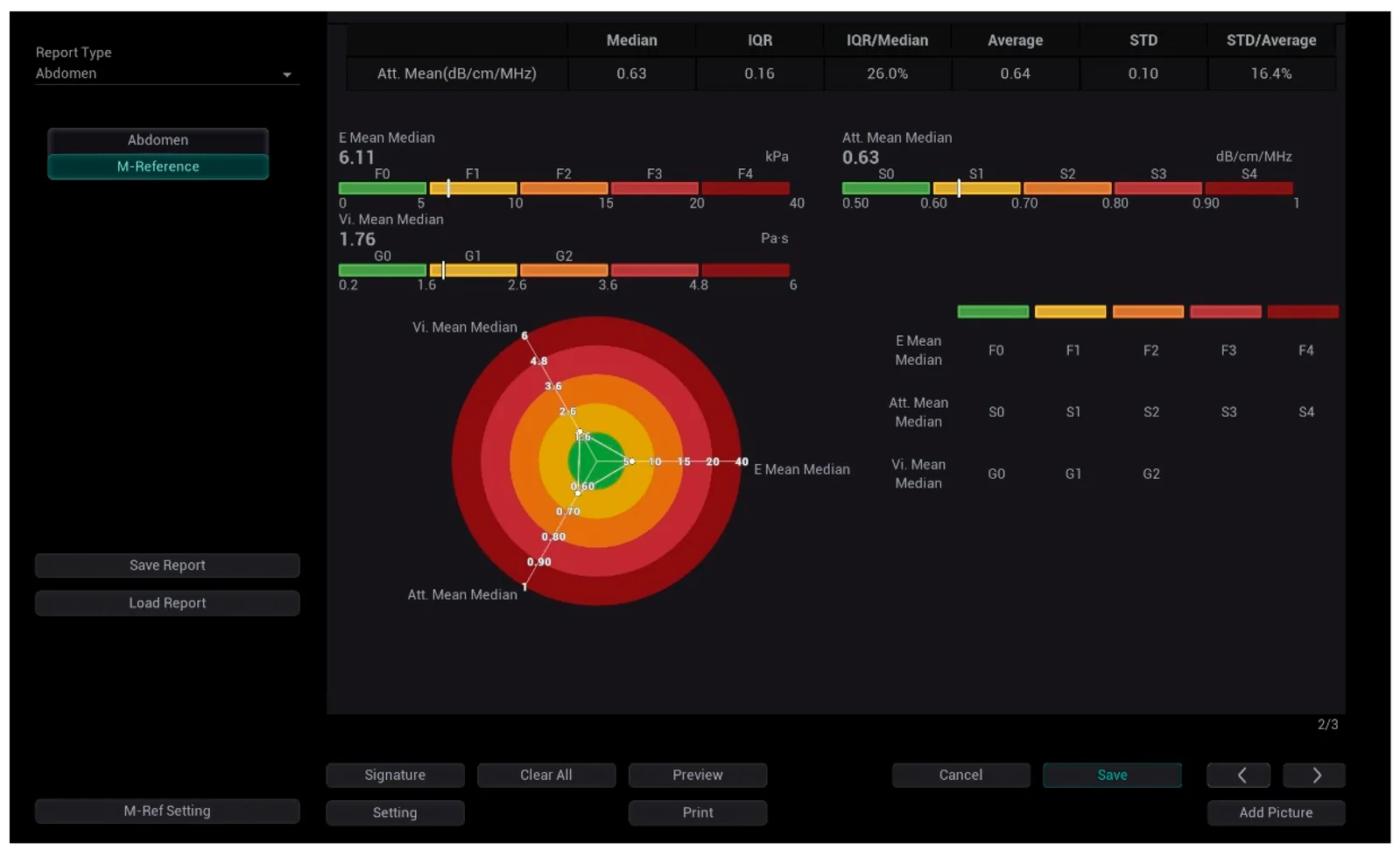
Multiparametric graphical report generated by the Acoustic Intelligence Technology platform. The report displays the median liver stiffness measurement (E), liver viscosity measurement (Vi), and ultrasound attenuation coefficient (Att.), together with their corresponding system-generated categories. E is expressed in kPa, Vi in Pa·s, and Att. in dB/cm/MHz. The color-coded concentric diagram provides a simultaneous visual representation of the three measured tissue properties.

**Figure 3:**
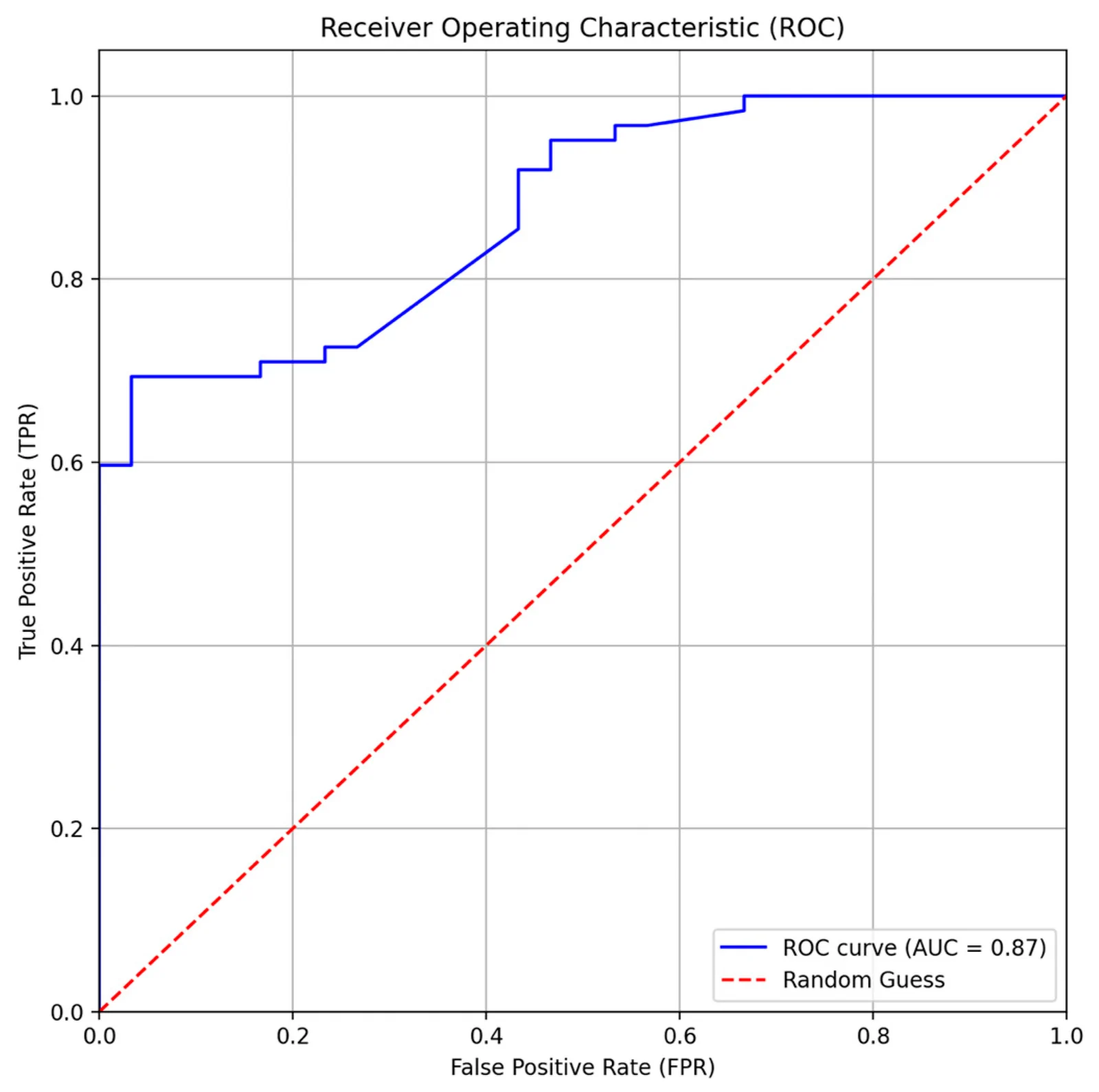
Receiver operating characteristic curve for discrimination between patients with alcohol-associated hepatitis and healthy controls.

**Figure 4:**
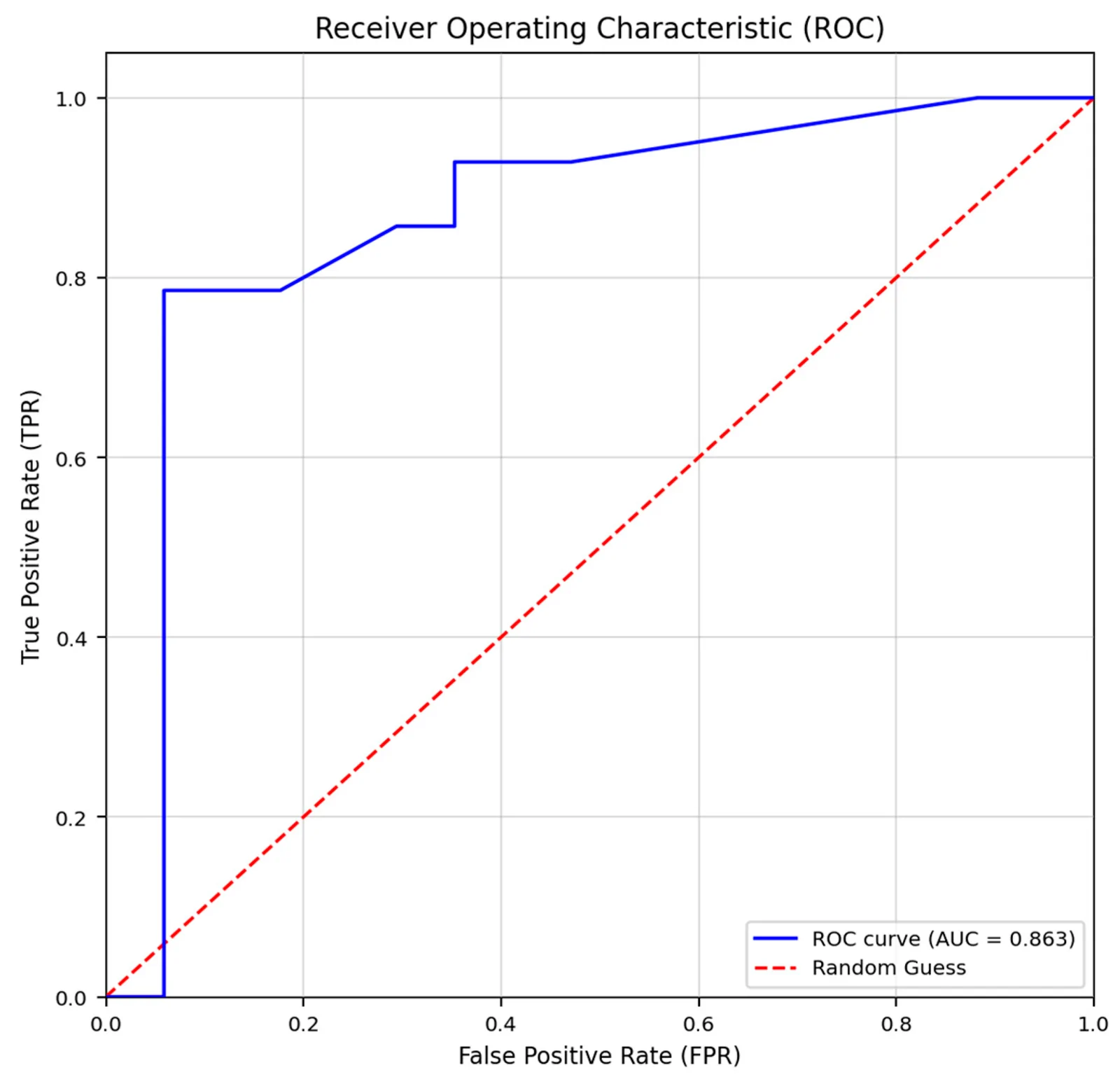
Receiver operating characteristic curve for discrimination of MDF-defined severe versus non-severe alcohol-associated hepatitis within the AH cohort.

**Table 1:** Clinical, laboratory, and imaging characteristics of patients with alcohol-associated hepatitis and healthy controls.

Variable	AH Group (n = 62)	Healthy Controls (n = 30)	*p*-Value
Liver stiffness, kPa	10.00 [8.00–15.50]	1.23 [1.14–5.00]	<0.001
Attenuation coefficient, dB/cm/MHz	0.78 [0.65–0.85]	0.60 [0.60–0.65]	<0.001
STVi viscosity-related parameter, Pa·s	2.50 [1.60–3.65]	1.40 [1.30–1.60]	<0.001
White blood cell count, ×10^3^/µL	8.00 [5.25–10.25]	6.10 [5.60–6.85]	0.067
C-reactive protein, mg/dL	4.00 [1.20–9.15]	0.40 [0.40–0.50]	<0.001
Erythrocyte sedimentation rate, mm/h	11.0 [5.5–29.5]	6.0 [4.5–8.0]	<0.001
Alanine aminotransferase, U/L	52.0 [43.5–65.0]	24.0 [21.0–30.0]	<0.001
Aspartate aminotransferase, U/L	65.0 [41.0–103.0]	19.0 [18.0–20.5]	<0.001
Total bilirubin, mg/dL	3.80 [3.00–8.70]	0.90 [0.80–1.02]	<0.001
International normalized ratio	1.50 [1.20–1.90]	0.90 [0.89–1.09]	<0.001
Prothrombin time, s	17.00 [14.10–19.40]	10.00 [9.90–11.00]	<0.001
Gamma-glutamyl transferase, U/L	129.0 [103.0–182.5]	33.0 [18.0–51.0]	<0.001
Alkaline phosphatase, U/L	100.0 [67.0–135.0]	58.0 [45.5–66.0]	<0.001

Note: Values are presented as median [interquartile range]. Between-group comparisons were performed using the Mann–Whitney U test. AH, alcohol-associated hepatitis.

**Table 2:** Imaging and laboratory characteristics according to MDF-defined AH severity.

Variable	Severe AH (n = 28)	Non-Severe AH (n = 34)	*p*-Value
Liver stiffness, kPa	15.50 [10.65–18.50]	9.50 [7.00–10.31]	<0.001
Attenuation coefficient, dB/cm/MHz	0.79 [0.70–0.82]	0.70 [0.63–0.88]	0.674
STVi viscosity-related parameter, Pa·s	3.65 [2.97–3.80]	1.60 [1.60–2.50]	<0.001
White blood cell count, ×10^3^/µL	7.51 [5.30–10.25]	9.00 [5.30–10.00]	0.557
C-reactive protein, mg/dL	6.00 [4.25–14.25]	1.40 [0.88–4.00]	<0.001
Erythrocyte sedimentation rate, mm/h	15.5 [9.0–40.2]	8.0 [5.0–11.0]	0.002
Alanine aminotransferase, U/L	53.5 [43.2–74.2]	50.0 [44.0–60.0]	0.519
Aspartate aminotransferase, U/L	71.0 [62.0–132.5]	55.0 [35.0–85.0]	0.004
Total bilirubin, mg/dL	7.85 [3.11–20.30]	3.00 [3.00–4.80]	0.002
International normalized ratio	1.95 [1.87–2.04]	1.20 [1.04–1.40]	<0.001
Prothrombin time, s	19.75 [18.62–21.00]	14.20 [11.30–15.20]	<0.001
Gamma-glutamyl transferase, U/L	132.5 [113.2–183.8]	111.0 [90.0–170.0]	0.096
Alkaline phosphatase, U/L	115.0 [78.0–195.0]	97.0 [45.8–120.0]	0.011

**Table 3:** Spearman correlations between the STVi viscosity-related parameter and imaging, inflammatory, biochemical, and severity parameters.

Variable	Spearman ρ	*p*-Value
Liver stiffness, kPa	0.67	<0.001
Attenuation coefficient, dB/cm/MHz	−0.08	0.516
White blood cell count, ×10^3^/µL	−0.05	0.704
C-reactive protein, mg/dL	0.63	<0.001
Erythrocyte sedimentation rate, mm/h	0.41	0.001
Alanine aminotransferase, U/L	0.07	0.595
Aspartate aminotransferase, U/L	0.36	0.004
Total bilirubin, mg/dL	0.16	0.217
International normalized ratio	0.63	<0.001
Prothrombin time, s	0.63	<0.001
Gamma-glutamyl transferase, U/L	0.26	0.042
Alkaline phosphatase, U/L	0.29	0.022
Maddrey discriminant function	0.66	<0.001

Note: The STVi viscosity-related parameter is expressed in Pa·s. ρ, Spearman rank correlation coefficient.

**Table 4:** Multivariable linear regression model of the STVi viscosity-related parameter.

Variable	B	HC3 Robust SE	95% CI	*p*-Value	VIF
Constant	0.849	0.233	0.384 to 1.315	<0.001	—
Liver stiffness, kPa	0.111	0.023	0.066 to 0.157	<0.001	1.01
C-reactive protein, mg/dL	0.062	0.011	0.040 to 0.085	<0.001	1.01

Note: The STVi viscosity-related parameter (Pa·s) was the dependent variable. B represents the unstandardized regression coefficient. HC3 heteroscedasticity-consistent standard errors are reported. Model R^2^ = 0.650; adjusted R^2^ = 0.638.

**Table 5:** Exploratory proof-of-concept performance of the STVi viscosity-related parameter for discrimination between patients with alcohol-associated hepatitis and healthy controls.

Performance Measure	Value	95% CI
AUC	0.873	0.797–0.934
Sensitivity	66.1%	52.0–77.6%
Specificity	96.6%	82.7–99.9%
Positive Predictive Value	97.6%	85.5–99.6%
Negative Predictive Value	58.0%	49.2–84.4%

**Table 6:** Exploratory performance of the STVi viscosity-related parameter for discrimination of MDF-defined severe alcohol-associated hepatitis.

Performance Measure	Value	95% CI
AUC	0.863	0.756–0.955
Sensitivity	78.6%	59.0–91.7%
Specificity	94.1%	80.3–99.3%
Positive Predictive Value	91.7%	73.0–99.0%
Negative Predictive Value	84.2%	68.7–94.0%

**Table 7:** Exploratory comparison of discriminatory performance for MDF-defined severe AH.

Parameter	AUC for MDF-Defined Severe AH
STVi viscosity-related parameter	0.863
Liver stiffness	0.758
C-reactive protein	0.809

## Data Availability

The individual participant data are not publicly available due to ethical and privacy restrictions. Data may be available from the corresponding author upon reasonable request, subject to institutional and ethical approval.

## Abbreviations

AH, alcohol-associated hepatitis; ALD, alcohol-associated liver disease; ALP, alkaline phosphatase; ALT, alanine aminotransferase; AST, aspartate aminotransferase; CRP, C-reactive protein; ESR, erythrocyte sedimentation rate; MDF, Maddrey discriminant function; ROI, region of interest; STVi, Sound Touch Visco-elastography Imaging.
